# Supplementary material for: Evolution of STAT2 resistance to flavivirus NS5 occurred multiple times despite genetic constraints
Source: Nat Commun. 2024 Jun 26;15:5426. doi: 10.1038/s41467-024-49758-0 (PMC11208600; doi:10.1038/s41467-024-49758-0)
Supplement: Supplementary file 1 — Supplementary Information [file 41467_2024_49758_MOESM1_ESM.pdf]

Supplementary Information

Evolution of STAT2 resistance to flavivirus NS5 occurred multiple times despite genetic constraints

Ethan C. Veit[1], Madihah S. Salim[1], Mariel J. Jung[1], R. Blake Richardson[1], Ian N. Boys[2,3], Meghan Quinlan[2,3], Erika A. Barrall[1], Eva Bednarski[1], Rachael Hamilton[1], Caroline Kikawa[4,5,6], Nels C. Elde[2,3], Adolfo García-Sastre[1,7,8,9], Matthew J. Evans[1,#]

[1]Department of Microbiology, Icahn School of Medicine at Mount Sinai, New York, NY, USA, [2]Department of Human Genetics, University of Utah, Salt Lake City, UT, USA, [3]Howard Hughes Medical Institute, Chevy Chase, MD, USA [4]Medical Scientist Training Program, University of Washington, Seattle, WA, USA, [5]Department of Genome Sciences, University of Washington, Seattle, WA, USA, [6]Basic Sciences, Fred Hutch Cancer Center, Seattle, WA, USA [7]Global Health and Emerging Pathogens Institute, Icahn School of Medicine at Mount Sinai, New York, NY, USA, [8]Department of Medicine, Division of Infectious Diseases, Icahn School of Medicine at Mount Sinai, New York, NY, USA, [9]The Tisch Cancer Institute, Icahn School of Medicine at Mount Sinai, New York, NY, USA,

[#]Corresponding author:
Matthew J. Evans, Ph.D.
Department of Microbiology
Icahn School of Medicine at Mount Sinai
1 Gustave L. Levy Place, Box 1124
New York, NY 10029
Tel: 212-241-7319
Fax: 212-534-1684
Email: matthew.evans@mssm.edu

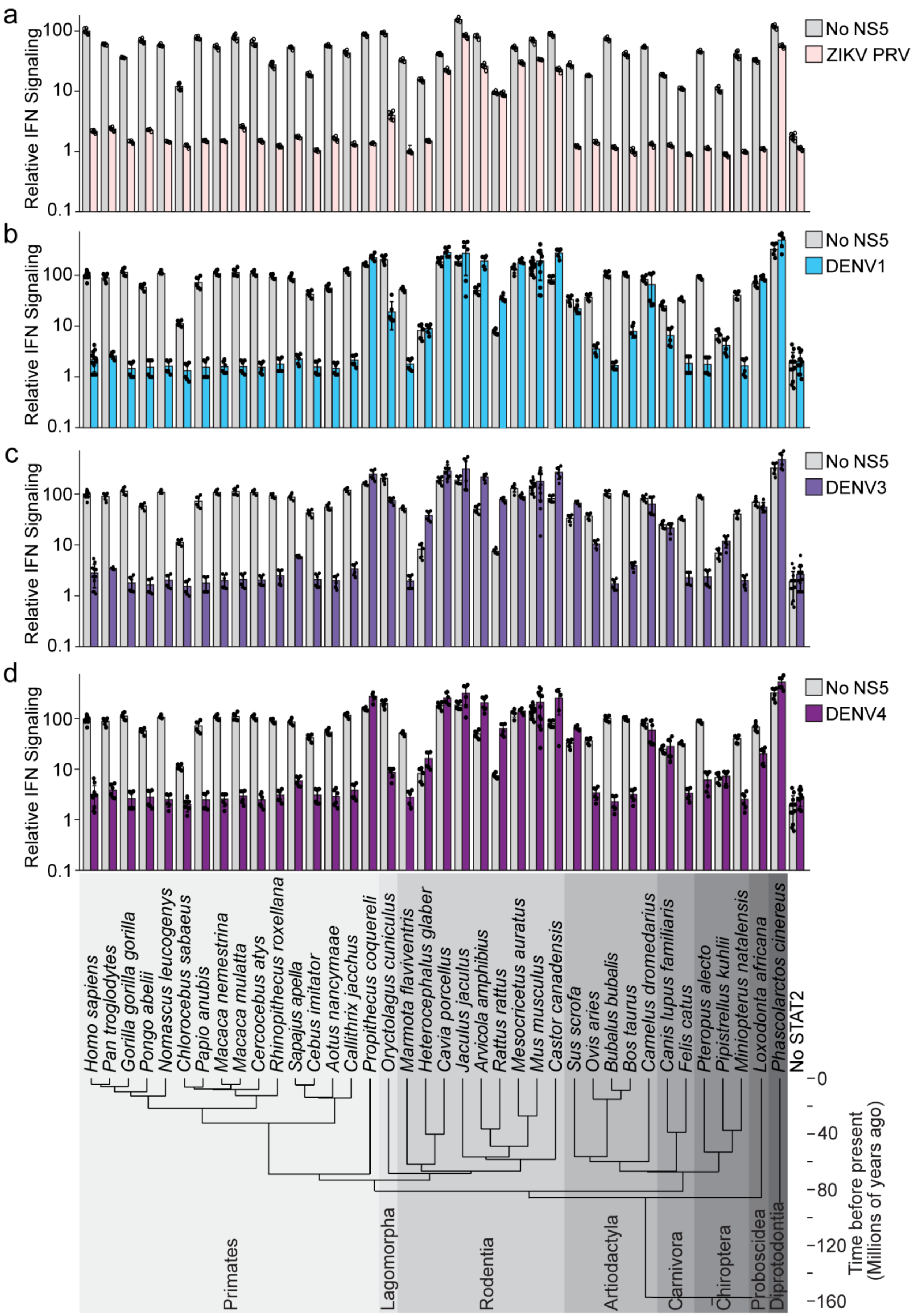
a
b
c
d
Relative IFN Signaling
100
10
1
0.1
No NS5
ZIKV PRV
DENV1
DENV3
DENV4
Homo sapiens
Pan troglodytes
Gorilla gorilla gorilla
Pongo abelii
Nomascus leucogenys
Chlorocebus sabaeus
Papio anubis
Macaca nemestrina
Macaca mulatta
Cercocebus atys
Rhinopithecus roxellana
Sapajus apella
Cebus imitator
Aotus nancymaae
Callithrix jacchus
Propithecus coquereli
Oryctolagus cuniculus
Marmota flaviventris
Heterocephalus glaber
Cavia porcellus
Jaculus jaculus
Arvicola amphibius
Rattus rattus
Mesocricetus auratus
Mus musculus
Castor canadensis
Sus scrofa
Ovis aries
Bubalus bubalis
Bos taurus
Camelus dromedarius
Canis lupus familiaris
Felis catus
Pteropus alecto
Pipistrellus kuhlii
Miniopterus natalensis
Loxodonta africana
Phascolarctos cinereus
No STAT2
Primates
Lagomorpha
Rodentia
Artiodactyla
Carnivora
Chiroptera
Proboscidea
Diprotodontia
Time before present (Millions of years ago)
0
40
80
120
160

Supplementary Fig. 1: Species Susceptibility to ZIKV PRV, DENV1, DENV3, and DENV4 NS5

Relative IFN signaling mediated by each species ND/CCD chimera in the absence of a viral antagonist (grey) and in the presence of **a**, ZIKV PRV NS5 (light pink; mean ± s.d., n=6), **b**, DENV1 NS5 (turquoise; mean ± s.d., for *Homo sapiens*, *Mus musculus*, and No STAT2, n=12, all others n=6), **c**, DENV3 NS5 (light purple; mean ± s.d., for *Homo sapiens*, *Mus musculus*, and No STAT2, n=12, all others n=6), and **d**, DENV4 NS5 (purple; mean ± s.d., for *Homo sapiens*, *Mus musculus*, and No STAT2, n=12, all others n=6). Source data are provided as a Source Data file. All IFN signaling values are derived from at least two independent experiments each with three technical replicates.

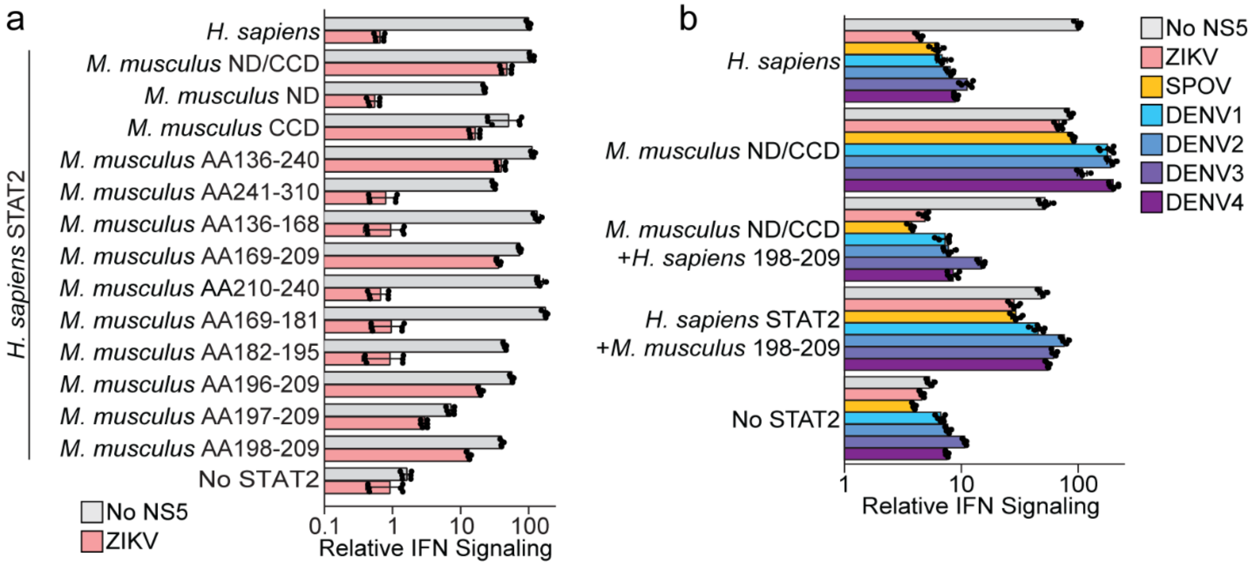

Supplementary Fig. 2: Mapping *M. musculus* STAT2 resistance determinants

a, Relative IFN signaling (mean ± s.d., n=6) mediated by each indicated *H. sapiens* STAT2 mutant with successively smaller regions of *M. musculus* STAT2 in the absence of a viral antagonist (grey) and in the presence of ZIKV NS5 (pink). STAT2 chimeras swapping either the ND or the CCD individually with the *M. musculus* sequence into *H. sapiens* STAT2 showed the *M. musculus* CCD alone conferred ZIKV NS5 resistance. Through further subchimeras of this region of *M. musculus* STAT2 we identified that AA 198-209 of *M. musculus* STAT2 was sufficient to confer ZIKV NS5 resistance to *H. sapiens* STAT2. **b**, (mean ± s.d., n=6) Relative IFN signaling mediated by each of indicated STAT2 mutant in the absence of an antagonist (grey), ZIKV NS5 (pink), SPOV NS5 (yellow), DENV1 NS5 (turquoise), DENV2 NS5 (blue), DENV3 NS5 (light purple), and DENV4 NS5 (purple). Source data are provided as a Source Data file. All IFN signaling values are derived from at least two independent experiments each with three technical replicates.

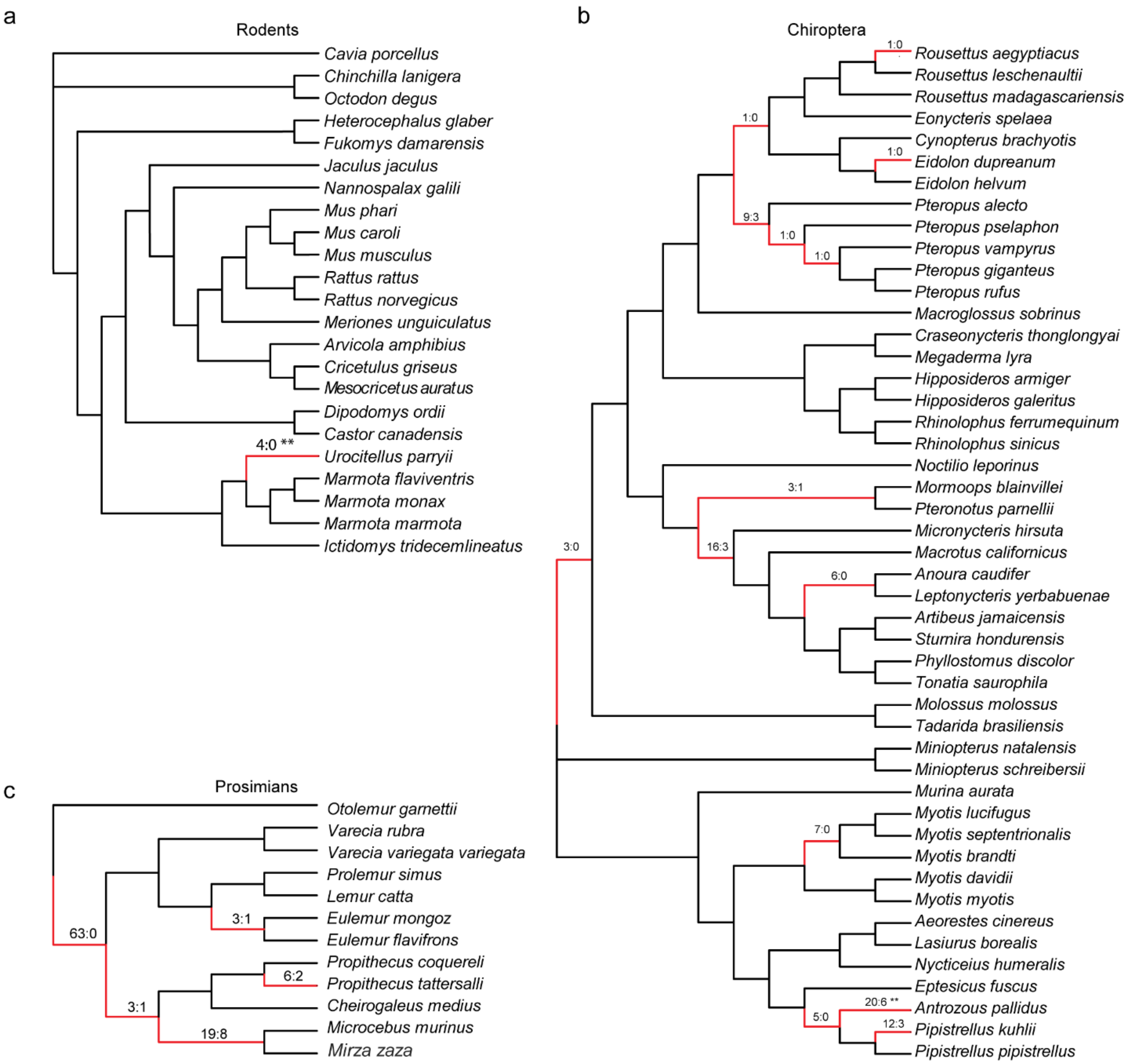

Supplementary Fig. 3: Branch-site analysis of STAT2 in mammalian lineages. a-c, Branch-site analysis using PAML for STAT2 in three mammalian lineages; Rodentia (**a**), Chiroptera (**b**), and Prosimians (**c**). Branches along which positive selection was detected are labeled in red, with the ration of nonsynonymous to synonymous substitutions above the given branch. Branches marked with (**) indicate additional detection of rapid evolution signatures using ABSREL.

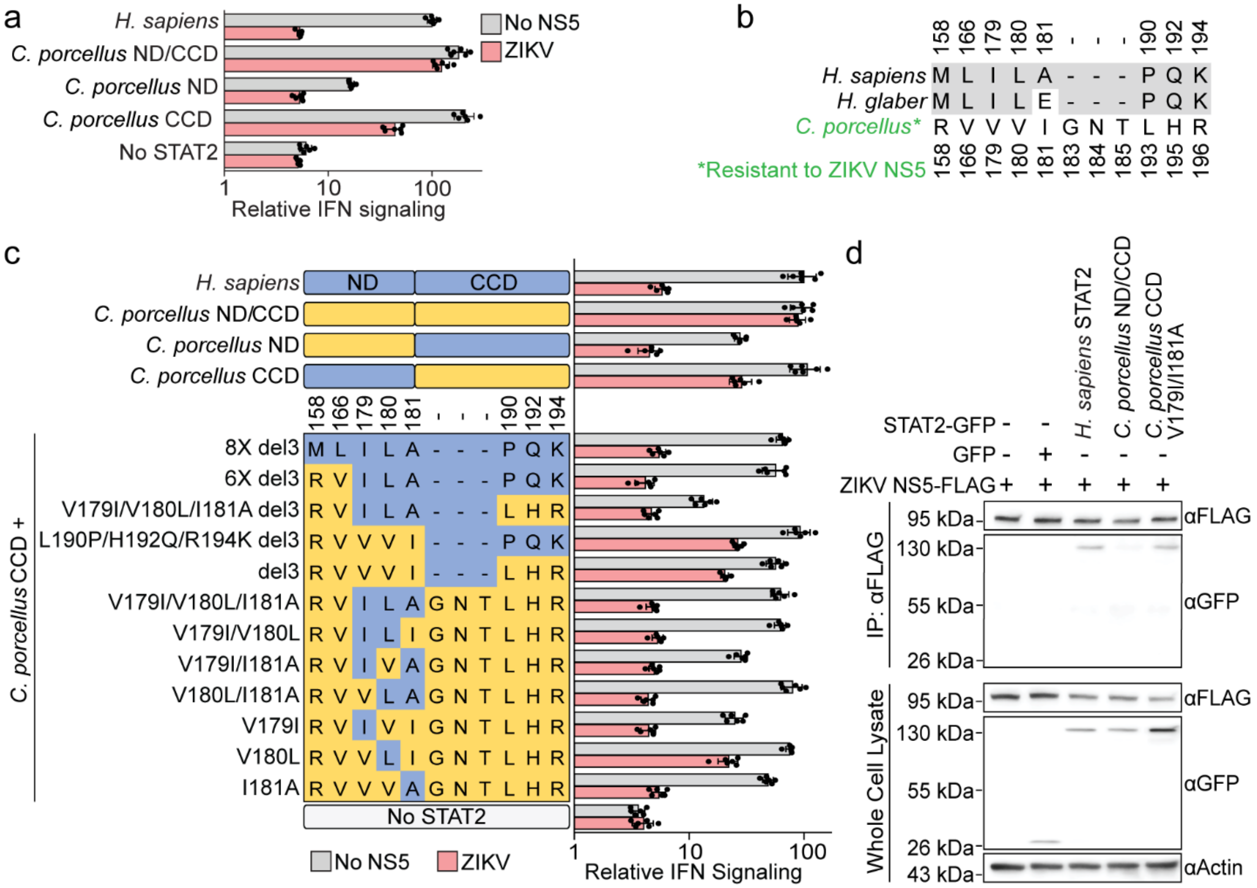

Supplementary Fig. 4: Mapping *C. porcellus* STAT2 resistance determinants

a, Relative IFN signaling (mean ± s.d., n=6) mediated by the indicated *H. sapiens* and *C. porcellus* STAT2 chimeras in the absence of a viral antagonist (grey) and in the presence of ZIKV NS5 (pink). Both the ND and CCD domains of *C. porcellus* STAT2 contribute to its resistance to ZIKV NS5 antagonism. **b**, To define *C. porcellus* STAT2 CCD residues that might contribute its resistance to ZIKV NS5, we compare *H. sapiens*, *H. glaber*, and *C. porcellus* STAT2 sequences. Based on the structure of ZIKV NS5 in complex with *H. sapiens* STAT2 (composite of PDB:6WCZ, 6UX2), we identified STAT2 residues which are unique among these species and make contacts with ZIKV NS5. Species with a STAT2 resistant to ZIKV NS5 antagonism marked in green (*). Numbering along the top corresponds to the *H. sapiens* sequence and the numbering along to bottom corresponds to the *C. porcellus* sequence. **c**, Relative IFN signaling (mean ± s.d., n=6) of

the various chimeras of *H. sapiens* and *C. porcellus* STAT2 sequence was measured in the absence (grey) and presence (pink) of ZIKV NS5. When the 11 amino acids listed in (b) were swapped to the *H. sapiens* sequence in the *C. porcellus* CCD chimera (“8X del3”), it rendered it fully susceptible to ZIKV NS5 antagonism. By generating various *C. porcellus* CCD chimeras with smaller subsets of these 11 amino acid swaps, we identified residues 179 and 181 as necessary for resistance to ZIKV NS5 antagonism as swapping any individually to the *H. sapiens* amino acid rendered it susceptible. **d**, Co-immunoprecipitation of *H. sapiens* STAT2, *C. porcellus* STAT2 ND/CCD chimera, and the *C. porcellus* ND/CCD STAT2 chimera bearing a subset of the mapped *C. porcellus* resistance determinants (*C. porcellus* ND, and residues 179, 181) swapped to the *H. sapiens* sequence. Whole cell lysates of STAT2 KO 293T cells expressing GFP-tagged STAT2, and FLAG-tagged ZIKV NS5 were mixed and STAT2 interaction was accessed via immunoblot following FLAG antibody immunoprecipitation of ZIKV NS5. While *H. sapiens* STAT2 is pulled down by immunoprecipitation of ZIKV NS5, the *C. porcellus* ND/CCD chimera does not interact. However, when the mapped resistance determinants are swapped to the *H. sapiens* sequence, interaction with ZIKV NS5 is restored. Source data are provided as a Source Data file. All IFN signaling values are derived from at least two independent experiments each with three technical replicates.

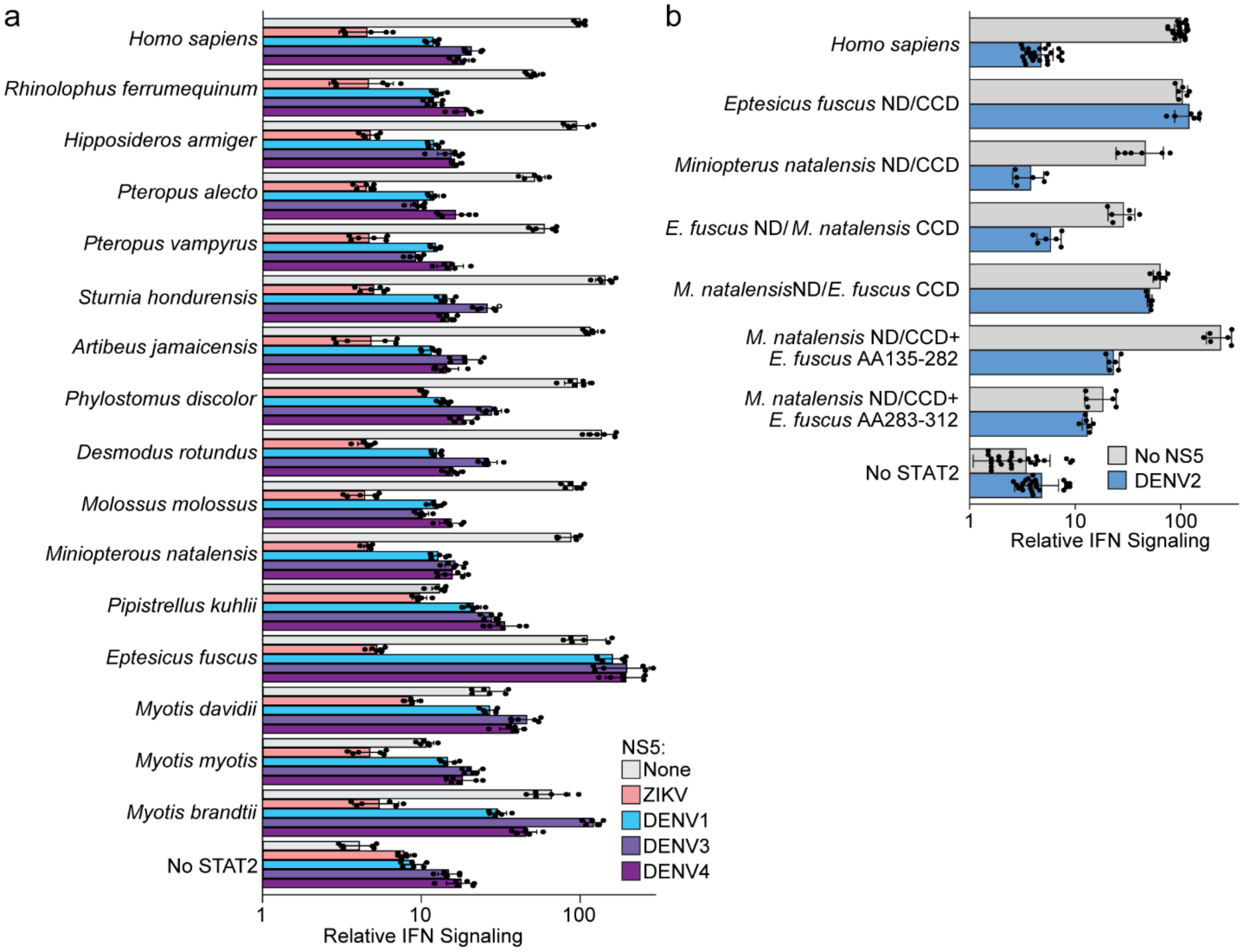

Supplementary Fig. 5: Evaluating DENV NS5 resistance in bats

a, Measuring susceptibility of Bat STAT2 proteins to flavivirus NS5 antagonism. Relative IFN signaling (mean ± s.d., n=6) of 15 bat species STAT2 chimeras in the absence of an antagonist (grey), ZIKV NS5 (pink), DENV1 NS5 (turquoise), DENV3 NS5 (light purple), and DENV4 NS5 (purple). While the signaling of all tested STAT2 proteins was reduced in the presence of ZIKV NS5, some species showed resistance to DENV NS5 antagonism. Consistent with the DENV2 NS5 data in (Fig. 4a) the STAT2 of the five species in the family Vespertilionidae, *Pipistrellus kuhlii*, *Eptesicus fuscus*, *Myotis davidii*, *Myotis myotis*, and *Myotis brandtii*, were resistant to antagonism by the NS5 protein from DENV1, DENV3, and DENV4, while all other bat species were susceptible. **b**, Relative IFN signaling (mean ± s.d., for *H. sapiens* and No STAT2 n=48, all

others n=12) of chimeras between the susceptible *Miniopterous natalensis* STAT2 with sequences from the resistant *E. fuscus* STAT2 in the absence (grey) and presence (blue) of DENV2 NS5. Chimeras swapping the ND domains between the resistant *E. fuscus* ND/CCD chimera and the susceptible *M. natalensis* ND/CCD chimera showed that the *E. fuscus* resistance determinants are in the CCD as the *E. fuscus* ND/*M. natalensis* CCD chimera is susceptible to DENV2 NS5 while the *M. natalensis* ND/*E. fuscus* CCD chimera is resistant. We further mapped the *E. fuscus* resistance determinants to the last 35 amino acids of the CCD which contains eight amino acid differences from *M. natalensis*. Having identified that the *E. fuscus* CCD conferred resistance we divided it into two regions: AA135-282 and AA283-317. The *M. natalensis* ND/CCD chimera with the sequence of *E. fuscus* STAT2 at AA135-282 (*M. natalensis* ND/CCD+*E. fuscus* AA135-282) exhibited was antagonized by DENV2 NS5. In contrast, the *M. natalensis* ND/CCD chimera with the sequence of *E. fuscus* STAT2 at AA283-317 (*M. natalensis* ND/CCD+*E. fuscus* AA283-317) was resistant to DENV2 NS5 antagonism. Source data are provided as a Source Data file. All IFN signaling values are derived from at least two independent experiments each with three technical replicates.

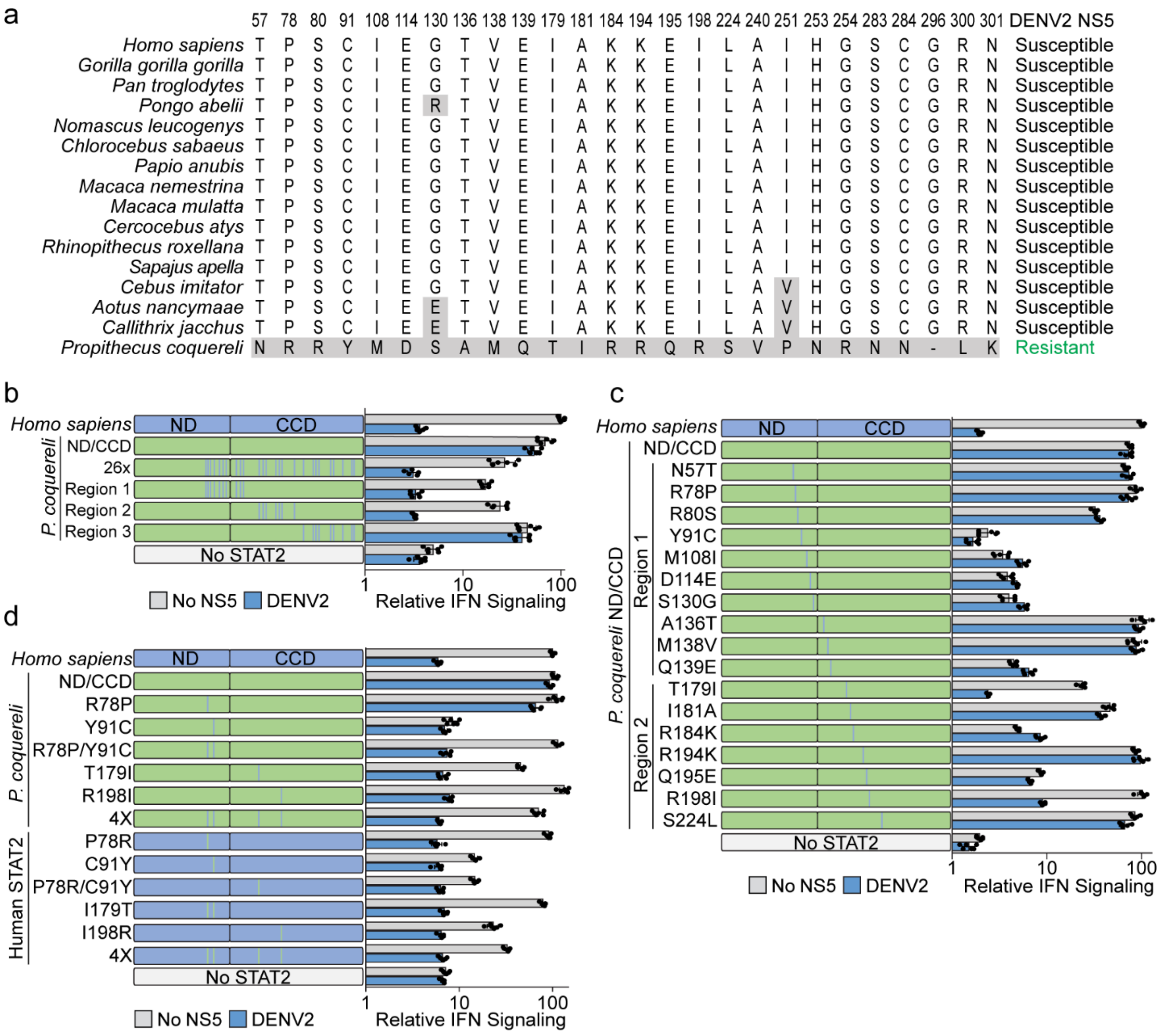

Supplementary Fig. 6: Mapping DENV NS5 resistance in *P. coquereli* STAT2

a, To map the *P. coquereli* STAT2 resistance determinants for DENV NS5 antagonism, we aligned the sequence of the 16 primate STAT2 sequences evaluated in Fig. 1. As *P. coquereli* was the only species among these primates that was resistant to DENV NS5 antagonism, we looked for residues that were unique in this species. Shown is the alignment of these 26 residues, 25 of which have a unique *P. coquereli* amino acid, and one has a single amino acid *P. coquereli* deletion. **b**, Mapping of the *P. coquereli* STAT2 resistance determinants to DENV NS5. Relative IFN signaling (mean ± s.d., n=6) of *P. coquereli* ND/CCD chimeras with various amino acid

changes in the absence (grey) and presence (blue) of DENV2 NS5. While the *P. coquereli* ND/CCD chimera is resistant to DENV 2 NS5, swapping the 26 residues identified in (a) to the *H. sapiens* sequence (*P. coquereli* ND/CCD 26x) renders it susceptible to antagonism. We further narrowed these 26 residues by dividing the ND/CCD into three regions. At least one residue in both “Region 1” and “Region 2” are necessary for resistance to DENV NS5 antagonism as swapping either of these regions renders it susceptible. **c**, Single amino acid mapping of the *P. coquereli* STAT2 resistance determinants to DENV NS5. Relative IFN signaling (mean ± s.d., n=6) of *P. coquereli* ND/CCD chimeras with various single amino acid changes in the two regions identified in (b), in the absence (grey) and presence (blue) of DENV2 NS5. We generated 17 *P. coquereli* ND/CCD chimeras each with one of the single amino acid changes contained within “Region 1” and “Region 2” evaluated in (b). Of the 10 amino acid changes contained within “Region 1” nine were able to induce the reporter to levels greater than when no STAT2 is added, but none had an impact of DENV NS5 resistance. One residue in this region (Y91C) was unable to induce reporter activity in the absence of an antagonist preventing us from accessing the impact of DENV2 NS5 resistance of this chimera. Of the seven changes contained within “Region 2”, two single amino acid swaps in the *P. coquereli* ND/CCD chimera all were able to induce reporter activity in the absence of an antagonist, and two (T179I and R198I) increased susceptibility to DENV NS5. **d**, Evaluation of reciprocal chimeras of the *P. coquereli* STAT2 resistance determinants to DENV2 NS5 between *H. sapiens* STAT2 and the *P. coquereli* ND/CCD chimera. Relative IFN signaling (mean ± s.d., n=6) activity of various *H. sapiens* STAT2 and *P. coquereli* ND/CCD chimeras in the absence (grey) and presence (blue) of DENV2 NS5. In (c) we identified that the R78P single amino acid change had little impact on DENV2 NS5 resistance and that the Y91C change greatly impaired signaling in the absence of an antagonist. Here we show that these two changes (R78P and Y91C) made at the same time in the *P. coquereli* ND/CCD chimera yields a STAT2 protein that is able to induce reporter activity in the absence of DENV2 NS5, but has lost the resistance seen in the WT *P. coquereli* ND/CCD chimera. Similarly, a making the 179I or

R198I changes individually in the *P. coquereli* ND/CCD chimera increases susceptibility to DENV2 NS5. The *P. coquereli* ND/CCD chimera with all four of these changes made at once (4x = R78P, Y91C, T179I, and R198I) was able to signal in the absence, but not in the presence of DENV2 NS5. No combination of the reciprocal changes for these four residues were able to increase resistance of *H. sapiens* STAT2 to DENV2 NS5, but some decrease signaling in the absence of an antagonist, although not to the level when no STAT2 was added. Source data are provided as a Source Data file. All IFN signaling values are derived from at least two independent experiments each with three technical replicates.

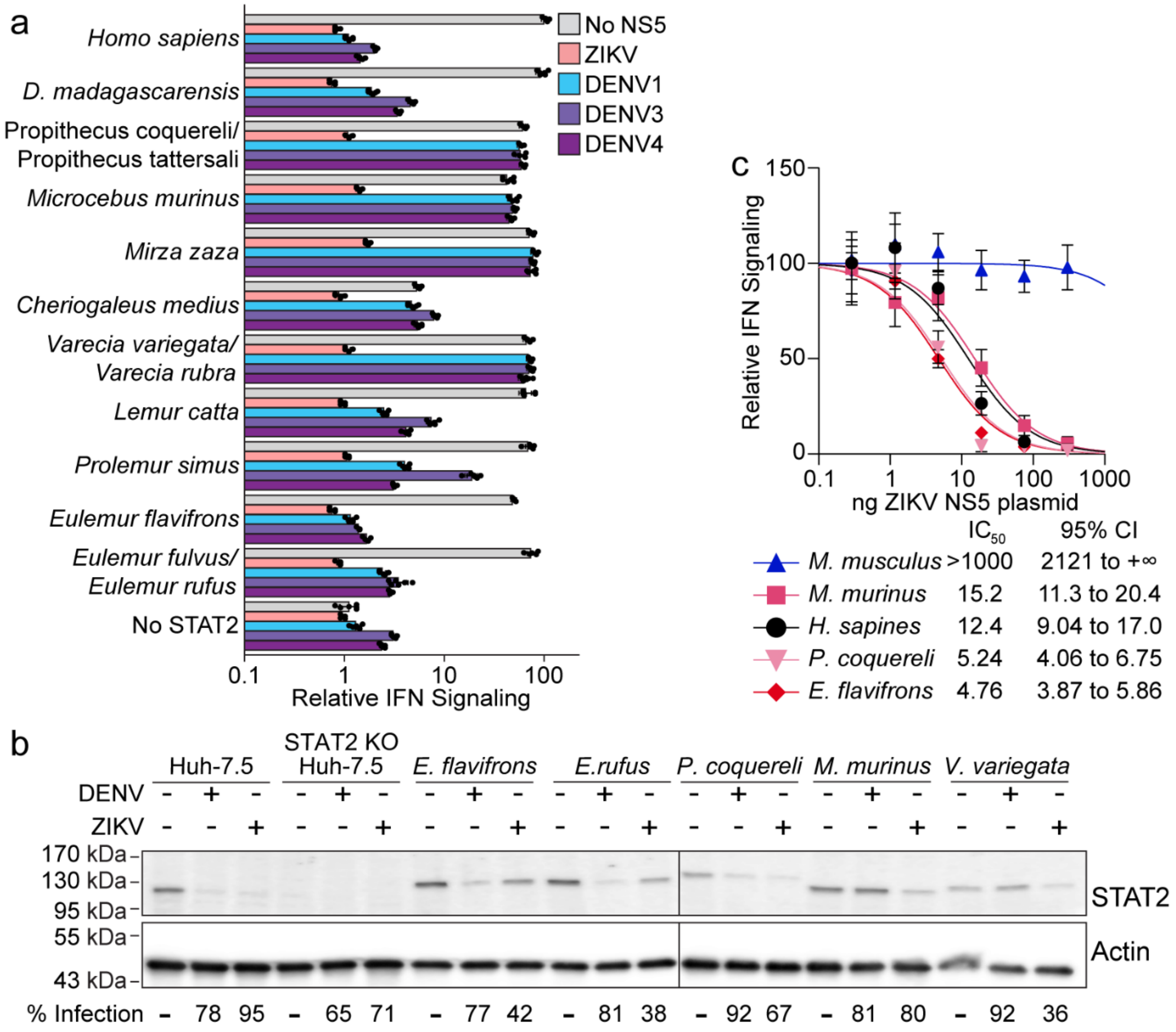

Supplementary Fig. 7: Mapping DENV NS5 resistance in *P. coquereli* STAT2

a, Analysis of the susceptibility of various lemur species to flavivirus NS5 antagonism. Relative IFN signaling (mean ± s.d., n=6) of ten unique STAT2 ND/CCD chimeras corresponding to 13 different lemur species STAT2 sequences in the absence of an antagonist (grey), and the presence of ZIKV NS5 (pink), DENV1 NS5 (turquoise), DENV3 NS5 (light purple), and DENV4 NS5 (purple). All were similarly susceptible to ZIKV NS5, but five ND/CCD chimeras corresponding to the STAT2 sequence from seven species were resistant to NS5 from DENV serotypes 1, 3, and 4, consistent with the resistance to DENV2 NS5 shown in (Fig. 5a). Four

species (*D. madagascarensis*, *Eulemur flavifrons*, *E.fulvus*, and *E. rufus*) had a STAT2 which was as susceptible to DENV NS5 antagonism as *H. sapiens* STAT2. STAT2 from *Lemur catta* and *Prolemur simus* exhibited intermediate susceptible to DENV NS5, with DENV3 NS5 being weakest antagonist. **b**, Western blot analysis of STAT2 protein levels in various *H. sapiens* and lemur cell lines 18 hours post infection with either DENV2 16681 or ZIKV MR766. Percent infection is indicated under each lane, which was used to normalize the amount of STAT2 degradation observed (Gating strategy and quantification in Supplementary Fig. 8). Images shown are a representative example of three independent experiments. **c**, Relative IFN signaling (mean ± s.d., n=6) of the STAT2 chimeras indicated in the legend in the presence of increasing doses of ZIKV NS5. Values are normalized to signaling in absence of ZIKV NS5 and the baseline is set to reporter levels in the absence of STAT2. Curves are fitted to the data using least-squares fit method of non-linear regression to calculate IC50 values for each STAT2 in units of ng of ZIKV NS5 plasmid. Source data are provided as a Source Data file. All IFN signaling values are derived from at least two independent experiments each with three technical replicates.

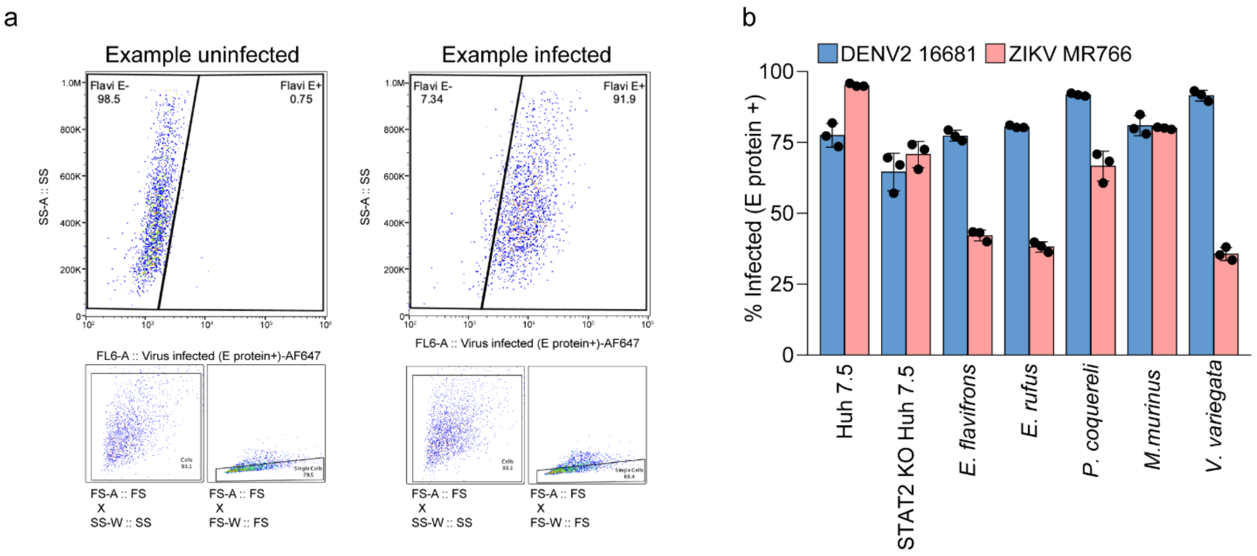

Supplementary Fig. 8: Infection Quantification for STAT2 degradation experiments. a, Gating strategy to quantify percent infection using intracellular staining with the α-Flavivirus E protein (4G2) antibody. One example each of for an uninfected control and a DENV infected sample. **b,** Using the gating strategy shown in (a), the percentage of infection for each cell type for both DENV (blue) and ZIKV (pink) infection was quantified. (mean ± s.d., n=3). These data were used to normalize the degree of STAT2 degradation measured in Fig. 5e and Supplementary Fig. 7b. Source data are provided as a Source Data file. All IFN signaling values are derived from at least two independent experiments each with three technical replicates.

	N	M7	M8	M8a	m7m8 LRT	m8m8a LRT	m7m8 P	m8m8a P	BEB m8	FUBAR	dN/dS	PAML	FUBAR
Prosimians	12	-2316.658005	-2311.243974	-2316.575414	10.828062	10.66288	0.004453651311	0.001093070628	5	9	0.52869	L80, K184, R254, K289, G290	K289, G290, L80, N51, R254, L150, K184, Y84, R153
Rodentia	23	-6672.952392	-6664.784429	-6667.622972	16.335926	5.677086	0.000283595112	0.017187891184	1	0	0.35201	R286	N/A
Chiroptera	47	-6788.095000	-6764.127000	-6779.030000	47.935900	29.806300	0.000000000039	0.000000048000	4	10	0.38508	P251, L258, Q286, P291	N53 L97 V127 P182 P251 E256 L253 Q286 T297 L301

Supplementary Table 1: Rapid evolution analysis of STAT2

Results of PAML and FUBAR for Prosimian, Rodents, and Bat STAT2 sequences. Individual residues identified to be rapidly evolving by each analysis are listed for each set of sequences analyzed. Residue numbering; Prosimians - "*Prolemur simus*", Rodentia - "*Mus musculus*", Chiroptera - "*Eptesicus fuscus*".
