# Supplementary material for: Evolution of STAT2 resistance to flavivirus NS5 occurred multiple times despite genetic constraints
Source: Nat Commun. 2024 Jun 26;15:5426. doi: 10.1038/s41467-024-49758-0 (PMC11208600; doi:10.1038/s41467-024-49758-0)
Supplement: Supplementary file 3 — Description of Additional Supplementary Files [file 41467_2024_49758_MOESM3_ESM.pdf]

Description of Additional Supplementary Files

File Name: Supplementary Data 1
Description: Results of FUBAR Analysis for species in the order Rodentia indicated in Supplementary Fig. 3a.

File Name: Supplementary Data 2
Description: Results of FUBAR Analysis for species in the Order Chiroptera indicated in Supplementary Fig. 3b.

File Name: Supplementary Data 3
Description: Results of FUBAR Analysis for prosimian species indicated in Supplementary Fig. 3c.

File Name: Supplementary Data 4
Description: List of STAT2 and flavivirus NS5 protein sequences used in this study.
