# Supplementary material for: Evolution of STAT2 resistance to flavivirus NS5 occurred multiple times despite genetic constraints
Source: Nat Commun. 2024 Jun 26;15:5426. doi: 10.1038/s41467-024-49758-0 (PMC11208600; doi:10.1038/s41467-024-49758-0)
Supplement: Supplementary file 8 — Reporting Summary [file 41467_2024_49758_MOESM8_ESM.pdf]

nature portfolio

Corresponding author(s): Matthew Evans

Last updated by author(s): May 15, 2024

Reporting Summary

Nature Portfolio wishes to improve the reproducibility of the work that we publish. This form provides structure for consistency and transparency in reporting. For further information on Nature Portfolio policies, see our Editorial Policies and the Editorial Policy Checklist.

Statistics

For all statistical analyses, confirm that the following items are present in the figure legend, table legend, main text, or Methods section.

n/a	Confirmed	
[ ]	[x]	The exact sample size (*n*) for each experimental group/condition, given as a discrete number and unit of measurement
[ ]	[x]	A statement on whether measurements were taken from distinct samples or whether the same sample was measured repeatedly
[ ]	[x]	The statistical test(s) used AND whether they are one- or two-sided<br>*Only common tests should be described solely by name; describe more complex techniques in the Methods section.*
[x]	[ ]	A description of all covariates tested
[ ]	[x]	A description of any assumptions or corrections, such as tests of normality and adjustment for multiple comparisons
[ ]	[x]	A full description of the statistical parameters including central tendency (e.g. means) or other basic estimates (e.g. regression coefficient) AND variation (e.g. standard deviation) or associated estimates of uncertainty (e.g. confidence intervals)
[ ]	[x]	For null hypothesis testing, the test statistic (e.g. *F*, *t*, *r*) with confidence intervals, effect sizes, degrees of freedom and *P* value noted<br>*Give P values as exact values whenever suitable.*
[x]	[ ]	For Bayesian analysis, information on the choice of priors and Markov chain Monte Carlo settings
[x]	[ ]	For hierarchical and complex designs, identification of the appropriate level for tests and full reporting of outcomes
[x]	[ ]	Estimates of effect sizes (e.g. Cohen's *d*, Pearson's *r*), indicating how they were calculated

Our web collection on statistics for biologists contains articles on many of the points above.

Software and code

Policy information about availability of computer code

Data collection	Gallios Flow Cytometer (Beckman Coulter)<br>BioTek Synergy H1 Multimode Reader (Agilent)
Data analysis	Flow data were analyzed by FlowJo (v10) and Kaluza Analysis<br>Species phylogeny were generated using Mesquite (Version 3.81)<br>Figures were generated using GraphPad Prism (V8) and Adobe Illustrator (v 28.3)<br>Rapid evolution analysis was conducted using PAML and FUBAR.<br>Sequence analysis and alignments preformed with MacVector (v 18)

For manuscripts utilizing custom algorithms or software that are central to the research but not yet described in published literature, software must be made available to editors and reviewers. We strongly encourage code deposition in a community repository (e.g. GitHub). See the Nature Portfolio guidelines for submitting code & software for further information.

Data

Policy information about availability of data

All manuscripts must include a data availability statement. This statement should provide the following information, where applicable:

- Accession codes, unique identifiers, or web links for publicly available datasets
- A description of any restrictions on data availability
- For clinical datasets or third party data, please ensure that the statement adheres to our policy

The STAT2 sequence data generated in this study have been deposited on NCBI Genbank (Cheirogaleus medius (PP600020), Varecia variegata (PP600021), Varecia rubra (PP600022), Propithecus tattersalli (PP600023), Mirza zaza (PP600024), Eulemur rufus (PP600025), Eulemur mongoz (PP600026), Eulemur flavifrons (PP600027), Daubentonia madagascariensis (PP600028)). Source data including unprocessed western blot images are provided with this paper. All biological materials including plasmids and cells lines will be made available for research purposes upon request

Research involving human participants, their data, or biological material

Policy information about studies with human participants or human data. See also policy information about sex, gender (identity/presentation), and sexual orientation and race, ethnicity and racism.

Reporting on sex and gender	N/A
Reporting on race, ethnicity, or other socially relevant groupings	N/A
Population characteristics	N/A
Recruitment	N/A
Ethics oversight	N/A

Note that full information on the approval of the study protocol must also be provided in the manuscript.

Field-specific reporting

Please select the one below that is the best fit for your research. If you are not sure, read the appropriate sections before making your selection.

☒ Life sciences ☐ Behavioural & social sciences ☐ Ecological, evolutionary & environmental sciences

For a reference copy of the document with all sections, see nature.com/documents/nr-reporting-summary-flat.pdf

Life sciences study design

All studies must disclose on these points even when the disclosure is negative.

Sample size	No statistical methods were utilized to predetermine sample size. Instead at least two independent experiments each with three biological replicates were performed, which is standard for the nature of the in vitro experiments used in this study.
Data exclusions	No data were excluded.
Replication	For the luciferase-based reporter assay, at least two independent experiments each with three biological replicates were performed. STAT2 degradation experiment was conducted three times. Co-immunoprecipitation experiments were performed twice.
Randomization	No randomization was utilized in this study and covariates were not controlled for as no statistical test that requires this was utilized in this study.
Blinding	Experiments were not blinded in this study the nature of the experiments did not permit this.

Reporting for specific materials, systems and methods

We require information from authors about some types of materials, experimental systems and methods used in many studies. Here, indicate whether each material, system or method listed is relevant to your study. If you are not sure if a list item applies to your research, read the appropriate section before selecting a response.

Materials & experimental systems

n/a	Involved in the study	
	☒	Antibodies
	☒	Eukaryotic cell lines
☒		Palaeontology and archaeology
☒		Animals and other organisms
☒		Clinical data
☒		Dual use research of concern
☒		Plants

Methods

n/a	Involved in the study	
☒		ChIP-seq
	☒	Flow cytometry
☒		MRI-based neuroimaging

Antibodies

Antibodies used

1. anti-β-Actin-HRP (Abcam, ab49900) (Dilution-1:20000)
2. anti-FLAG M2-HRP (Sigma-Aldrich, A8592) (Dilution-1:10000)
3. anti-GFP antibody (Abcam, ab290) (Dilution-1:1000)
4. anti-rabbit IgG-HRP (Amersham, NA934V) (Dilution-1:10000)
5. anti-flavivirus E (Absolute antibody, Ab00230-10.0) (Dilution-1:1000)
6. anti-STAT2 antibody (Invitrogen, #44-362G) (Dilution-1:1000)
7. anti-human IgG-AF647 (Invitrogen, A56019) (Dilution-1:1000)

Validation

1. anti-β-Actin-HRP (Abcam, ab49900) was verified by Abcam for western blot. (https://www.abcam.com/products/primary-antibodies/hrp-beta-actin-antibody-ac-15-ab49900.html?productWallTab=ShowAll)
2. anti-FLAG M2-HRP (Sigma-Aldrich, A8592) was verified by Sigma-Aldrich for or western blotting (https://www.sigmaaldrich.com/US/en/product/sigma/a8592#product-documentation)
3. anti-GFP antibody (Abcam, ab290) was verified by Abcam for western blot. (https://www.abcam.com/products/primary-antibodies/gfp-antibody-ab290.html)
4. anti-rabbit IgG-HRP (Amersham, NA934V) was verified by Fisher Scientific for western blot. (https://www.fishersci.com/shop/products/anti-rabbit-igg-peroxidase-linked-species-specific-whole-antibody-from-donkey-secondary-antibody-cytiva/45001276)
5. anti-flavivirus E (Absolute antibody, Ab00230-10.0) was verified by Absolute antibody for flow cytometry. (https://absoluteantibody.com/product/anti-flavivirus-group-antigen-d1-4g2-4-15-4g2/)
6. anti-STAT2 antibody (Invitrogen, #44-362G) was verified by Invitrogen for western blot. (https://www.thermofisher.com/antibody/product/STAT2-Antibody-Polyclonal/44-362G)
7. anti-human IgG-AF647 (Invitrogen, A56019) was verified by Invitrogen for Flow cytometry. (https://www.thermofisher.com/antibody/product/Goat-anti-Human-IgG-H-L-Secondary-Antibody-Recombinant-Polyclonal/A56019)

Eukaryotic cell lines

Policy information about cell lines and Sex and Gender in Research

Cell line source(s)

HEK-293T cells (ATCC, CRL-3216)
STAT2 KO HEK-293T cells (derived in this study)
Huh 7.5 cells (provided by Charles Rice, Rockefeller University, New York, NY)
Primary lemur fibroblasts (derived in this study from tissue samples)
-Propithecus Coquereli (Unknown Sex)
-Microcebus murinus (Female)
-Eulemur flavifrons (Unknown Sex)
-Eulemur rufus (Female)

Authentication

HEK-293T and Huh 7.5 cells have been authenticated using Short Tandem Repeat (STR) profiling.

Mycoplasma contamination

We continuously monitor every 10th passage or 4-month of use for mycoplasma contamination. Cells used in this study tested negative for mycoplasma.

Commonly misidentified lines
(See ICLAC register)

No commonly misidentified cell lines were used in this study.

Plants

Seed stocks	N/A
Novel plant genotypes	N/A
Authentication	N/A

Flow Cytometry

Plots

Confirm that:

- [x] The axis labels state the marker and fluorochrome used (e.g. CD4-FITC).
- [x] The axis scales are clearly visible. Include numbers along axes only for bottom left plot of group (a 'group' is an analysis of identical markers).
- [x] All plots are contour plots with outliers or pseudocolor plots.
- [x] A numerical value for number of cells or percentage (with statistics) is provided.

Methodology

Sample preparation	Adherent cells were collected by trypsinization and fixed in 1% PFA for 1 hour prior to antibody staining. For intracellular staining, cell membrane were permeabilized by the addition of 0.1% saponin to wash buffer and antibody dilutions.
Instrument	Data collected on Beckman Coulter Gallios Flow Cytometer.
Software	Data were analyzed using Kaluza Analysis Software and FlowJo v10.
Cell population abundance	For experiments quantifying percent infection, greater than 2000 total cells were analyzed per sample. After sorting out debris and gating on single cells 2000 cells remained to quantify percent infection.
Gating strategy	Debris was removed using FS-A by SS-W gating and doublets were excluded using FS-A by FS-W. To quantify percent infection, negative and positive gates were drawn using a stained, uninfected control sample.

- [x] Tick this box to confirm that a figure exemplifying the gating strategy is provided in the Supplementary Information.
